# Structure–Elastic Properties Relationships in Gelling Carrageenans

**DOI:** 10.3390/polym13234120

**Published:** 2021-11-26

**Authors:** Loïc Hilliou

**Affiliations:** Institute for Polymers and Composites (IPC), University of Minho, Campus de Azurém, 5800-048 Guimarães, Portugal; loic@dep.uminho.pt

**Keywords:** carrageenan, hydrogel, nonlinear elasticity

## Abstract

Gelling carrageenans are polysaccharides extracted from the Gigartinales order of red algae. These are additives used essentially in the food industry for texturizing, stabilizing or gelling various formulations. Although a consensual gel mechanism has been reached which encompasses a coil-to-helix transition followed by the self-assembling of helices in a network, the structure–elastic relationships in the network are still to be clearly established. This paper reviews the reports in which carrageenan gel structures have been systematically compared with gel elastic properties. The focus is on the sizes documented for structural units, such as strands, aggregates, voids or network meshes, as well as on the reported linear and nonlinear elastic characteristics. The insufficient rationalization of carrageenan gel elasticity by models which take on board mechanically relevant structural features is underlined. After introducing selected linear and nonlinear elastic models, preliminary results comparing such models to structural and rheological data are presented. In particular, the concentration scaling of the strain hardening exhibited by two types of carrageenan gels is discussed.

## 1. Introduction

Carrageenans are a family of sulfated polysaccharides isolated from a specific order of red algae, namely, the Gigartinales. In spite of a controversy which started two decades ago about their food safety [1], carrageenans are extensively used in the food industry, and more recently in cosmetics and pharmaceutics [2,3,4]. At high enough polysaccharide concentrations in water and in the presence of cations, hot carrageenan solutions form hydrogels upon cooling. The commercial relevance of carrageenans is owed to such thermo-reversible gelling abilities. Carrageenans are thus seen as promising candidates, for instance, for replacing gelatin in pharmaceutical applications [5]. 

**Figure 1:**
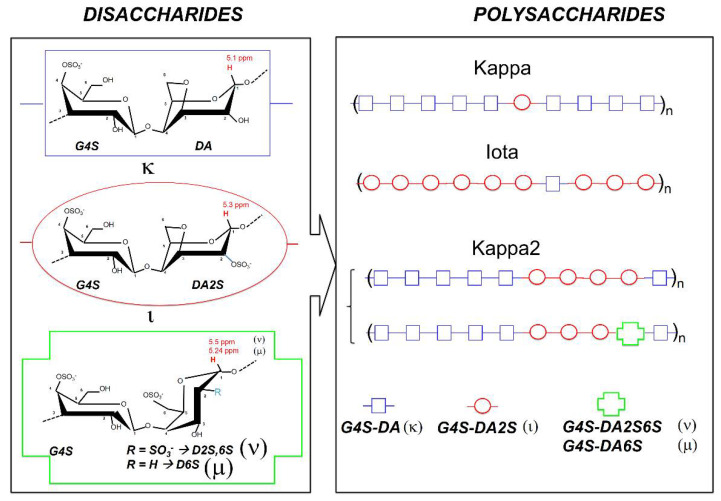
Chemical structures of industrially relevant gelling carrageenans.

Carrageenans are natural linear polyelectrolytes built from repeating disaccharide units made of 3-linked β-D-galactopyranose (labelled as ***G***-units), and 4-linked α-D-galactopyranose (***D***-units) or 4-linked 3,6-anhydrogalactose (***A***-units). Figure 1 presents the chemical structures of the gelling carrageenans that are most utilized commercially. Iota-carrageenan (Iota) is nearly a homopolymer of ***G4S-DA2S*** disaccharide units, containing a very low amount (of the order of 5 mol%) of ***G4S-DA*** disaccharide units [6]. Kappa-carrageenan (Kappa) is a little more heterogeneous, with up to 10 mol% of ***G4S-DA2S*** disaccharide units discontinuing blocks of ***G4S-DA*** disaccharide units [6]. A third gelling carrageenan, commercially known as kappa-2 or weak kappa (Kappa2), has progressively gained industrial impetus. Kappa2 replaces mixtures of Kappa and Iota in niche applications where intermediate gelling properties between the hard and brittle gels formed by Kappa, and the softer but deformable gels formed by Iota, are needed [7]. Kappa2 is a random block copolymer made of sequences of ***G4S-DA*** (making up 45–80 mol% of the hybrid chain [7]) and ***G4S-DA2S*** [8,9]. Depending on the seaweed and the extraction route used to isolate the polysaccharide, ***G4S-DA*** and ***G4S-DA2S*** blocks can be separated by more sulfated disaccharide units, such as ***G4S-D6S*** (mu-carrageenan) and ***G4S-D2S,6S*** (nu-carrageenan) [6,10]. 

**Figure 2:**
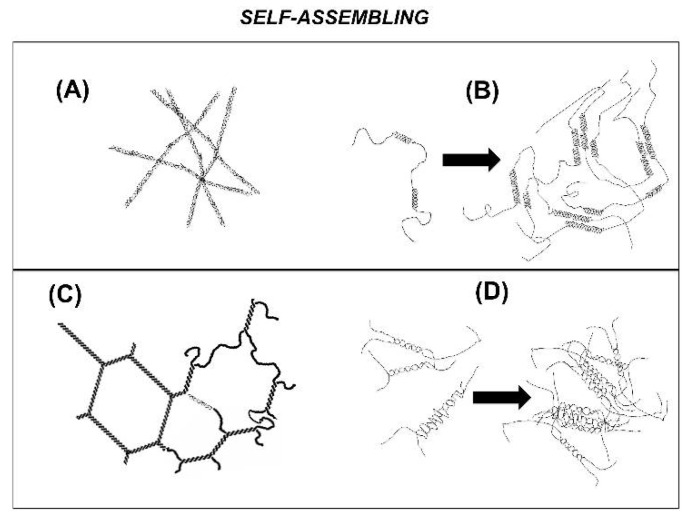
The most popular models of the self-assembling of carrageenan single helices (**A**,**B**) or double helices (**C**,**D**) giving rise to a three dimensional network showing elasticity: (**A**,**C**) represent self-assembling at the helical (or secondary structure) level, whereas (**B**,**D**) represent self-assembling at the super-helical level (tertiary or quaternary structures).

Although a gel mechanism, which encompasses a coil-to-helix transition followed by the self-assembling of carrageenan helices resulting in a three-dimensional network, is widely acknowledged in the literature, the network structure giving rise to elasticity is far less consensual among authors. First, the nature of the self-assembling helices is still a source of debate, as studies reporting the formation of single helices formed by a single carrageenan chain (see e.g., [11,12]) are opposed to studies supporting the formation of double helices formed by the intertwining of two carrageenan chains (see e.g., [13,14]) or the intramolecular cycling or hair pinning of a single carrageenan chain [15,16]. Second, many models have been drawn to describe the structure of the network built from the self-assembling of single or double helices. Figure 2 displays sketches of the most popular proposed networks. If one leaves aside the model of randomly packed rod-like polymers (Sketch A in Figure 2), which actually explains on a theoretical basis the network’s elasticity without the need for crosslinks between rods (see [17] and Section 3, below), all other sketches do not explicitly identify how stress is transmitted within the network. In the nested single helices model [11] (see Sketch B in Figure 1), and in the domain model [18] (Sketch D in Figure 2), it is not clear whether elasticity is ruled by the crosslinks made of nested single helices in B and domains of double helices in D, or by the carrageenan chains in non-helical conformation which bridge the different crosslinks. Ambiguity equally lies in the simpler chicken wire model [13] (see Sketch C in Figure 2), which convincingly represents a space panning network, but fails to identify whether double helices or splitting chains rule the network’s elasticity.

In his comprehensive review on gelling carrageenans [19], Piculell compiled the compelling evidence of networking at the super-helical level for Kappa gels (sketches B and D), giving hard gels in contrast to softer Iota gels where networking might occur at the helical level (sketches A and C). Since then, a large number of works have focused on the gel mechanism or the chemical structure–functional properties relationships, as recently reviewed elsewhere [20,21]. Fewer studies tried to systematically compare the carrageenan network structure with the corresponding gel elastic properties, with a view to extracting structure–elasticity relationships. The purpose of this paper is to review the recent progress made in identifying such relationships. The aim is to show that there is a lack of rationalization of the structural and elastic data by theories developed to describe the particular elasticity of filamentous networks. The latter have been used with some success for explaining structure–rheological properties relationships in various biopolymer and polymer gels [22,23,24] which share structural and elastic similarities with carrageenan gels.

The paper is organized as follows. Studies where carrageenan gel structures have been systematically compared with gel elastic properties will be shortly reviewed over the last two decades in the second section. It will be shown there that reports on the nonlinear rheological properties of carrageenan gels are still critically missing in the literature, as highlighted more than a decade ago by van de Velde [25]. Theories describing the elasticity of networks made of semi-flexible filaments are shortly reviewed in the third section. Thus, the significance of the nonlinear elastic properties for identifying structural features in these networks will be highlighted. Then, preliminary nonlinear rheological data for Kappa, Iota and Kappa2 gels will be presented. These results show a concentration scaling in the strain hardening behavior of Iota and Kappa2 gels. The strain hardening will be analyzed with the theories reviewed in Section 3 with a view to extracting the elastically relevant structural features to be compared with the structural information reviewed in Section 2.

## 2. Structure and Elastic Properties of Carrageenan Gels

Table 1 and Table 2 summarize the structural and elastic properties of Kappa and Iota gels, respectively, reported in studies from the last two decades attempting to identify the structure–elasticity relationships. Thus, all papers reporting only elastic properties or only documenting the structure of carrageenan gels are not listed in these tables. This is because the variety in the chemical structure of commercial carrageenans (disaccharide purity and counter-ions, as noted below) and in the employed gelling conditions virtually rule out any possible comparison between various publications. 

Though many studies used commercial samples as received, most of the results listed here were achieved after performing a purification step in order to produce a polyelectrolyte with a single type of counter-ion. This is of paramount importance for Kappa, which is known to show specific gel properties depending on the type of salt used to form the gels [26,27]. In contrast to this, it has been established that Iota does not exhibit such cation sensitivity unless a significant number of ***G4S-DA*** units remains present as impurities in Iota [28]. Thus, Table 1 and Table 2 report the details of the carrageenan samples used when available, namely, the type of cations used to gel the carrageenan and the type of carrageenan. 

**Table 1:** Structural and elastic properties of Kappa gels prepared with salt conditions and carrageenan samples specified in the first column “System”. Mesh size *ε*, length *L* and diameter *d* of strands were obtained from electronic microscopy (EM), fluorescence recovery after photobleaching (FRAP), particles tracking (PT), atomic force microscopy (AFM), nuclear magnetic resonance (NMR), small angle X-ray scattering (SAXS) or wide angle X-ray scattering (WAXD). Column *G*_0_ reports the range of gel linear shear storage modulus *G*_0_ measured at temperatures between 10 and 25 °C. The last column lists the referenced literature for the corresponding row.

Sample		Structural Features			Rheology	Ref.
Carrageean Form ^1^	Salt	*ε* (nm)	*L* (nm)	*d* (nm)	*G*_0_ (kPa)	
Com.	No salt	500–10^4^ (EM ^3^)	5 × 10^4^ (EM ^3^)	100–500 (EM ^3^)	0.02–20	[29]
Com.	No salt	2 × 10^3^–10^4^ (EM ^3^)	3 × 10^3^–10^4^ (EM ^3^)	2.34 (SAXS)	2.6	[30]
Na	CaCl_2_	Up to 5 × 10^4^ (EM ^3^)	Up to 5 × 10^4^ (EM ^3^)	10^2^–10^3^ (EM ^3^)	0.5–3.5	[31]
Na	NaCl	6 (WAXD)	>100 (AFM ^3^)	1.1 (AFM)	0.1	[32]
K	KCl	4.3 and 6 (WAXD)	>100 (AFM ^3^)	1.5 (AFM)	1.4	[32]
Na	KCl	<10 (NMR ^4^)	n.a. ^2^	n.a. ^2^	40	[33]
Na	KCL	<80 (FRAP)	n.a. ^2^	n.a. ^2^	1	[34]
Na	CaCl_2_	<80 (FRAP)	n.a. ^2^	n.a. ^2^	0.01	[34]
Na	KCl	<100 (PT)	n.a. ^2^	n.a. ^2^	2	[35]
Com.	KCl	9.5 (SAXS)	n.a. ^2^	25.1 (SAXS)	11	[36]
Com.	CaCl_2_	17.7 (SAXS)	n.a. ^2^	41 (SAXS)	130	[36]
Na	KCl	n.a. ^2^	<200 (AFM ^3^)	1.5–2 (AFM)	2	[16]
Na	CaCl_2_	n.a. ^2^	<500 (AFM ^3^)	1–1.5 (AFM)	0.2	[16]

^1^ Com.: commercial carrageenan used as received; Na: sodium form; K: potassium form; Ca: calcium form. ^2^ n.a.: non available. ^3^ Estimates from pictures reported in the corresponding reference. ^4^ Estimate from the hydrodynamic radius of the polymer probe used in NMR.

**Table 2:** Elastic and structural properties of Iota gels. Same column labelling and notes as in Table 1.

Sample		Structural Features			Rheology	Ref.
Carrageenan Form ^1^	Salt	ε (nm)	*L* (nm)	*d* (nm)	*G*_0_ (Pa)	
Na	NaCl	6 (WAXD)	<100 (AFM ^3^)	2 (AFM)	15	[32]
K	KCl	6 (WAXD)	<100 (AFM ^3^)	2 (AFM)	100	[32]
Na	KCl	>>10 (NMR) ^4^	n.a. ^2^	n.a. ^2^	400	[33]
Na	KCl	<90 (FRAP)	n.a. ^2^	n.a. ^2^	20	[34]
Na	CaCl_2_	<90 (FRAP)	n.a. ^2^	n.a. ^2^	80	[34]
Na	KCl	>>100 (PT)	n.a. ^2^	n.a. ^2^	100	[35]
Com.	KCl	n.a. ^2^	n.a. ^2^	14–62 (SAXS)	500	[36]
Com.	CaCl_2_	n.a. ^2^	n.a. ^2^	7–48 (SAXS)	310	[36]
Na	NaCl	n.a. ^2^	<200 (AFM ^3^)	1 (AFM)	10	[16]
Na	KCl	n.a. ^2^	<200 (AFM ^3^)	0.8 (AFM)	18	[16]
Na	CaCl_2_	n.a. ^2^	>10^3^ (AFM ^3^)	1 (AFM)	18	[16]
Com.	NaCl	1.2–1.5 nm (SAXS) 2 × 10^4^–3 × 10^4^ (EM)	27.5 (SAXS)	7.5 (SAXS)	130–160	[37]
Com.	No salt	<2 × 10^3^ (EM ^3^)	<2 × 10^3^ (EM ^3^)	10^2^–10^3^ (EM ^3^)	270	[38]

^1^ Com.: commercial carrageenan used as received; Na: sodium form; K: potassium form; Ca: calcium form. ^2^ n.a.: non available. ^3^ Estimates from pictures reported in the corresponding reference. ^4^ Estimate from the hydrodynamic radius of the polymer probe used in NMR.

Gel structural features, such as the mesh of the network *ε* (or the pore size), the length *L* and diameter *d* of strands or filaments, measured by microscopy of scattering techniques, are reported in Table 1 and Table 2. These features were either computed by the authors or inferred in the present study from the reported microscopic pictures. Data compiled in Table 1 and Table 2 essentially underline that electronic microscopy (EM) conveys microscopic structural information in contrast to other scattering or microscopic techniques which probe much smaller length scales. This is because cavities made by ice crystals formed during samples preparation are actually imaged by EM [39]. As such, EM can only be used for the qualitative comparison of structures within the same set of experiments. For instance, the addition of cations was shown to induce a coarser network in Kappa [31] and iota [37] gels, resulting in a drop in gel elasticity. 

More important, the quantitative information reviewed here suggests that minor structural differences are seen between Kappa and Iota gels. Small angle X-ray scattering (SAXS) suggests that *ε* varies from a few nanometers for Iota gels to more than ten nanometers for Kappa gels. There is, however, more variation in the reported values for *d*—between 2 and 41 nm for Kappa, and between 7.5 and 62 nm for Iota. These quantitative discrepancies stem from the variety of models used to interpret the intensity profiles, as well as from the diversity of carrageenan samples and gelling conditions. Note, however, that some reported *d* values are much larger than those returned by atomic force microscopy (AFM). AFM pictures show only very small radii of the order of 1 or 2 nm, which is consistent with *d* values documented by small angle neutron scattering of Kappa gels [22,40] and SAXS data for both Kappa and Iota gels [41].Clearly, each technique requires a specific sample preparation which impacts on the results, though a recent study established that, under certain conditions, X-ray scattering and transmission electron microscopy (TEM) return identical structural information for Kappa gels on length scales larger than 20 nm [42]. All documented AFM images suggest that *L* is of the same order of magnitude for both Iota and Kappa gels. The visual inspection of reported images suggests that *L* varies between 100 and 200 nm or thereabouts. This is in good agreement with values inferred from transmission electron microscopy of Kappa gelled in the presence of various salts [19,26], where rod-like filaments (superstrands) with lengths ranging from 100 to 400 nm were imaged. Note that one report gives a smaller length *L* for an Iota gel, as model fitting of the X-ray scattering intensity profile suggested a length of 27.5 nm [37].

All studies listed in Table 1 and Table 2 used rotational rheometry to extract the linear shear elastic modulus *G*_0_ of gels measured with small amplitude oscillatory shear. Data compiled in Table 1 and Table 2 confirm that overall Kappa forms gels which are one order of magnitude more elastic than Iota gels. Only a few works listed in Table 1 and Table 2 presented the large strain behavior of carrageenan gels. The strain for onset of nonlinear behavior *γ_NL_* was reported to depend on the gelling conditions (both salt and carrageenan concentrations), varying between 10% [30] and 1% [32] for Kappa, and between 15% [32] and 100% [38] for Iota. This again confirms the well documented brittleness of Kappa gels when compared with more strain-resistant Iota gels. However, none of these studies commented on the qualitative hardening or softening of gels under large strains. Similarly, the concentration dependence of *G*_0_ is virtually ignored in the set of references compiled in Table 1 and Table 2. A single work reported on the power law scaling *G*_0_~*c^n^* where *c* is the carrageenan concentration in the gels. An exponent *n* = 2.7 was found for Kappa gels [29]. Such scaling being of theoretical relevance, it has, however, been studied in dedicated rheological research (see Section 3, below). Gels are usually formed in the shearing geometry of the apparatus, and the gelling conditions were different from those used to gel the samples for structural characterization. This brings additional complexity for the identification of structure–elasticity relationships, since both properties are known to depend on the gelling route (see e.g., [10,26]). Structure–elasticity relationships in Kappa2 gels have not received much attention in the open literature [10], in contrast to the study of relationships in gelled mixtures of Kappa and Iota [20,33,43]. Section 4, below, will start to fill this gap of knowledge. 

Overall, the picture that emerges from the data reviewed in Table 1 and Table 2 is that large differences in Iota and Kappa gel elasticities are difficult to reconcile with their quite similar structures essentially consisting in semi-flexible filaments with length *L* of the order of 100 nm and thickness *d* of the order of 10 nm, arranged in a dense network with mesh size *ε* of the order of nanometers or tens of nanometers. Structural heterogeneity, where different types of filaments coexist together with different aggregates of filaments, has long been reported in carrageenan gels [19]. Structural non-homogeneity on length scales larger than 100 nm and with correlations over microns was recently highlighted with confocal scanning laser microscopy in Kappa gels, showing significant turbidity in contrast to homogeneous and clear Iota gels [43]. However, both gels showed nearly similar *G*_0_ values. Aggregates of filaments and further clustering of aggregates were also reported recently for Kappa [42]. Similar clustering was also reported for Iota gels thereby explaining their weaker elasticity when compared to Kappa gels [35]. Thus, cluster elasticity, as well as elasticity associated with cluster–cluster interactions, could also play a role in gels rheology, as it is in colloidal systems [44,45]. Returning to the macromolecular scale, dynamic heterogeneity has recently been highlighted, as free carrageenan chains with larger mobility in the solvent [33] can be released from the Iota or Kappa gel matrix [34]. However, the impact of such free chains on the gels’ elasticity remains unclear. Differences in the persistence length of Kappa and Iota filaments has also been inferred from AFM imaging [12,32]. The effect of cations on the persistence length of Kappa filaments was earlier suggested by EM pictures which related the presence of more flexible filaments with the weaker elasticity of the gels [26]. As explained below, in the theoretical section, the persistence length affects the rigidity of the corresponding filament and thus has a direct impact on the network’s elasticity.

## 3. Theoretical Analysis of the Elasticity of a Network of Semi-flexible Filaments

Table 3 lists selected constitutive equations which have been used to model the elastic properties of networks of semi-flexible filaments (also labelled as wormlike chains). Such networks exhibit strain hardening at large shear strains: upon leaving the linear regime of elasticity, the elastic shear modulus *G* increases in a nonlinear fashion with the increasing shear strain *γ*. The equations *G*(*γ*) describing the shear strain dependence of the network’s elasticity can be sorted in three categories. 

The first set of equations (Equations (1a), (2a) and (3a) in Table 3) are built on continuum theories where ad hoc expressions have been introduced for the nonlinear expansion of shear stress as a function of the shear strain. Thus, the origin of strain hardening differs depending on the choice for the nonlinear expansion. In Equation (1a), the finite extensibility $\frac{l_{max}}{l_{0}}$ of strands bridging two crosslinks in the network is responsible for the strain hardening described by an exponential function. This equation was successfully fitted to rheological data for physically associating block copolymer gels, but also to data of biological gels, such as actin [46]. In Equation (2a), the strain hardening lies in the fractal dimension *d_f_* of strands between two crosslinks [47], whereas the starting point for the strain expansion of the shear modulus is the phenomenological Blatz–Sharda–Tschoegl equation [48]. Equation (2a) was used with some success to reproduce the strain hardening of gelatin gels. More important, it incorporates the concentration dependence or the elastic shear modulus at small strain, *G*_0_ (see Equations (2c) and (2d)). The latter are scaling models originally proposed by Jones and Marques [49] to describe networks of rod-like polymers with constant crosslink functionality (only strand length is allowed to vary). These scaling laws were claimed to give a good account for carrageenan gels if one considers pure rod strands with *d_f_* = 1 [49]. In addition to Equation (2a), Groot et al. [47] also tested another molecular model where the strain hardening stems from the specific geometrical aspects of the flexible chains connected to rod-like strands making up the network (see Equation (3a)). This phenomenological model thus describes a certain degree of structural heterogeneity in the gel. 

**Table 3:** Theoretical predictions for the shear elastic modulus *G* and its nonlinear dependence *G*(*γ*) with shear strain *γ*. *G*_0_ is the (linear) elastic shear modulus at small strain. References to the original papers where the *G*(*γ*) expressions have been first introduced are gathered in the last column labelled “Ref.”.

Equations	*G*(*γ*)	Structural Parameters	Refs.
(1a) (1b)	$G\left(\gamma \right)=G_{0}\exp\left({\left(\gamma /\gamma^{*}\right)}^{2}\right)$ $\gamma^{*}=\lambda_{max}-{\left(\lambda_{max}\right)}^{-1},\;\lambda_{max}=\frac{l_{max}}{l_{0}}$	$\gamma^{*}$: Critical value of shear strain at which stiffening is dominant; *l_max_*: Length of a fully extended strand; *l*_0_: Length of a strand at rest.	[46]
(2a) (2b)	$G\left(\gamma \right)=\frac{2G_{0}}{n_{BST}}\frac{\lambda^{n_{BST}}-\lambda^{-n_{BST}}}{\lambda^{2}-\lambda^{-2}}$ $\lambda =\frac{1}{2}\gamma +\sqrt{1+\frac{1}{4}\gamma^{2}},\;n_{BST}=\frac{d_{f}}{d_{f}-1}$	*d_f_*: Fractal dimension of strands making up the network; *c*: Polymer volume fraction; Enthalpic elasticity: strands are connected by rigid (frozen) crosslinks [50]; Entropic elasticity: strands are connected by mobile crosslinks [50].	[47,49]
(2c)	$G_{0}\sim c^{\frac{3+d_{f}}{3-d_{f}}}$ enthalpic		
(2d)	$G_{0}\sim c^{\frac{3}{3-d_{f}}}$ entropic		
(3a) (3b) (3c)	$G\left(\gamma \right)=G_{0}A+G_{0}B\;\frac{\gamma^{2}}{1+0.125\gamma^{2}}$ $A=1.12+0.24\frac{l}{R_{0}}+0.068{\left(\frac{l}{R_{0}}\right)}^{2.5}$ $B=0.095+0.15\frac{l}{R_{0}}+0.114{\left(\frac{l}{R_{0}}\right)}^{2.5}$	*l*: Length of a rod-like strand connected to swollen chains in good solvent with radius of gyration *R*_0_.	[47]
(4a)	$G\left(\gamma \right)=\frac{G_{0}}{3}\left(1+2{\left(1-\beta \frac{\gamma^{2}+3}{3}\right)}^{-2}\right)$	*β*: Chain elongation ratio given by $\beta =\frac{\langle R_{in}{}^{2}\rangle }{R_{max}{}^{2}}$, where $\langle R_{in}{}^{2}\rangle$ is the mean-square average end-to-end distance of a strand in the unstrained network and $R_{max}{}^{2}$ is the square of the end-to-end distance of the fully extended strand; *E_bend_*: Bending rigidity of a strand; *c*: Polymer volume fraction;	[50]
(4b)	$G_{0}\sim E_{bend}c^{2}$		
(5)	$G\left(\gamma \right)=\frac{2nkT}{3}x^{2}\left(\frac{1-x^{4}}{e\pi {\left[1-\left(2+\gamma^{2}\right)x^{2}+x^{4}\right]}^{2}}-e\pi^{2}\right)$	*n*: Crosslink density in the network; *k*: Boltzmann constant; *T*: Temperature; *e*: Dimensionless strand stiffness parameter comparing bending and thermal energy; $x=\frac{\varepsilon }{L_{c}}$, where *ε* is the distance between crosslinks, and *L_c_* is the contour length of a strand joining the crosslinks.	[51]
(6a)	$G\left(\gamma \right)=\frac{G_{0}}{2}{\left[1-{\left(1+\frac{1}{2}\gamma \left(\gamma -\sqrt{\gamma^{2}+4}\right)\right)}^{\frac{1}{2}}\right]}^{5}\times \frac{\left(\gamma^{2}+2\right)\sqrt{\gamma^{2}+4}-\gamma }{{\left[\gamma +\frac{\left(\gamma -\sqrt{4+\gamma^{2}}\right)\gamma^{2}}{2}\right]}^{1/2}}$	*E_bend_*: bending rigidity of a strand with diameter *d*; *c*: polymer volume fraction.	[17]
(6b)	$G_{0}\sim \frac{E_{bend}}{d^{4}}c^{5}$		

The starting point for the second set of Equations (4a) and (5) is the nonlinear force–strain relationship describing the finite extensibility of wormlike chains with bending rigidity. Thus, the latter is the source for the strain hardening, together with the amount of pre-stress applied on the wormlike chain by the crosslinking. In Equation (4a), the nonlinearity is embedded in a single parameter, *β*, which indicates the stretch ability of a wormlike chain connecting two crosslinks in the network [50]. Thus, this model shares some similarities with Equation (1), since the same structural feature in the network is accounted for (compare the definitions of *β* and *λ_max_* in Table 3). Note that for stiff chains (rod-like strands), this theory indicates that the network modulus, *G*_0_, depends on the bending elasticity of the chains and shows a quadratic scaling with the polymer concentration (Equation (4b)), which is a special case of an enthalpic network with *d_f_* = 1 (see Equation (2c)). Equation (5) builds on two network characteristics to describe the strain hardening [51]. One relates to the topology of the network. This is described by the ratio *x* of the distance between two crosslinks (i.e., the mesh size of the network whose topology is modeled by a cubic structure where each edge crosslinks three wormlike chains) over the contour length of the wormlike chain joining the two crosslinks. The other network characteristic relates to a stiffness parameter *e* which balances the contribution of chain bending and thermal elasticity. Equations (4a) and (5) successfully described the strain hardening of collagen, actin or fibrin networks [50,51]. However, the rationalization of the network’s elasticity by two parameters, as in Equation (5), offers the possibility of predicting the first normal stress difference *N*_1_ of sheared networks. 

Indeed, when both *e* and *x* are large enough, which corresponds to a network with a mesh size of the order of the contour length of stiff enough filaments, *N*_1_ is found to be negative. This is in contrast with a finer network of more flexible strands which exhibits a positive *N*_1_ under shear [51].

The last equation in Table 3 stands out from the two latter sets of theories since no crosslinks are considered. Network elasticity originates from the rod-like character of the chains which are crowding the space. Under deformation, contacts between rods are increasing in such a way that more force is needed to induce further deformation [17]. As such, the strain hardening does not depend on any structural or elastic feature of the rod-like chains (see Equation (6a)), in contrast to the linear elastic shear modulus at small strain *G*_0_, which also presents a strong dependence with the polymer concentration (see Equation (6b)).

The nonlinear elasticity of carrageenan gels has nearly been overlooked, as outlined in Table 1 and Table 2. This is in contrast with studies reporting on the concentration dependence of *G*_0_. Table 4 gives a partial account of such studies performed with rotational rheometry, where power law relationships were documented or identified after re-plotting and fitting the data to a power law equation. 

Table 4 first indicates striking variability in the measured exponents *n* within the same type of carrageenan. This again connects to the natural variability in the chemical purity of Kappa and Iota samples and highlights the effectiveness of using different cations to modulate their gel elasticity to suite many applications [19]. Second, all exponents in Table 4 lie within the range of possible values predicted from the scaling relationships listed in Table 3. A minimum value of *n* = 1.5 is found if one takes strands as pure rods (*d_f_* = 1) and the elasticity of the network as purely entropic (Equation (2d)). On the other limit, taking *d_f_* = 2.5 for the fractal dimension of chains in an incipient gel [58] and an enthalpic network (Equation (2c)), one reaches *n* = 11. Nonetheless, the majority of the rheological studies compiled in Table 4 suggests that 2≤n≤3, which is in harmony with an enthalpic network of rod-like filaments (Equations (2c) and (4b)) with bending rigidity ruling the network’s elasticity. This was expected from the *L* and *d* values listed in Table 1 and Table 2. Clearly, testing of the nonlinear elastic properties is needed to extract more structural information than simply *d_f_* and also validate the consistency of a theoretical treatment of a rheological data set within a carrageenan concentration range. Such an exercise is presented in the following sections.

## 4. Linear–Nonlinear Elastic Properties of COMMERCIAL KAPPA and Iota and of a Selected Kappa2

Commercial Kappa and Iota (lots 0001432063 and 110M1861V, respectively, from Sigma-Aldrich, Darmstadt, Germany) were used as purchased. Whereas proton NMR spectroscopy could not detect any impurity in the Kappa sample, 8 mol% ***G4S-DA*** was detected in the commercial Iota. The carrageenan datasheets indicate that K^+^ cations are predominantly present in these commercial samples. However, gels formed by Kappa in KCl salts are prone to significant water syneresis [59], inherently leading to rheometrical issues. Kappa and Iota gels were thus prepared in 0.1 M NaCl by mixing the corresponding amount of carrageenan with 0.1 M NaCl at 80 °C for 30 min. Note that under such salt conditions nearly similar intrinsic viscosities were measured for both Kappa and Iota [60]. Kappa2 was selected from a series of hybrid carrageenans extracted from *Mastocarpus stellatus* seaweeeds [61]. This sample was isolated in the Na^+^ form, and the copolymer chain is made of 51.2 mol% of ***G4S-DA***, 31.7 mol% of ***G4S-DA2S*** and 17.1 mol% of non-gelling carrageenan disaccharide (mu- and nu-carrageenans). Though this extract showed the best gelling properties [61], it requires more than 3 wt.% Kappa2 to give gels with sufficient elasticity in 0.1 NaCl to allow rheological testing. Thus, with a view to testing a wider range of Kappa2 concentrations, Kappa2 gels were formed in 0.1 M KCl. 

**Figure 3:**
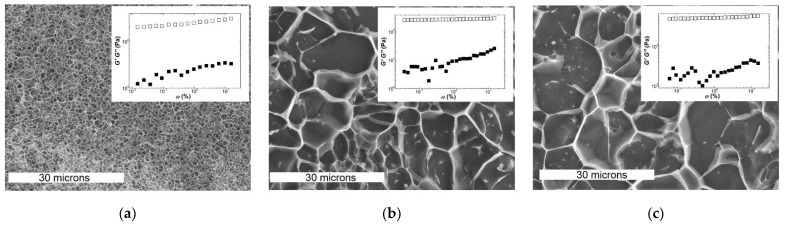
CryoSEM pictures and mechanical spectra (insets) of gels formed with 2 wt.% carrageenan at 20 °C for: (**a**) Kappa in 0.1 NaCl; (**b**) Iota in 0.1 NaCl; (**c**) Kappa2 in 0.1 KCl. White bars in CryoSEM pictures indicate 30 microns.

The experimental protocol used elsewhere to study the nonlinear rheology of Kappa2 gels by Fourier transform rheology was reproduced here [62]. Hot carrageenan solutions were loaded in the serrated plate–plate geometry (with 1 mm gap) of a strain-controlled rotational rheometer (ARES, TA Instruments, New Castle, DE, USA). This shearing geometry was pre-heated at 70 °C and covered with paraffin oil after sample loading to avoid evaporation. Serrated plates were used to limit wall slip as much as possible, as inferred from the low values of the stress second harmonics measured on-line during testing (see [62] for experimental detail). Carrageenan solutions were then cooled down to 20 °C and kept at this temperature for 3 h. This duration was sufficient to obtain gels with an equilibrated structure for all concentrations tested. Selected mechanical spectra recorded with a strain amplitude of 0.1% after 3 h are shown in Figure 3. The storage modulus *G*′ shows a very weak frequency dependence, with no upturn at the lowest measured frequency, thus confirming that gels were at equilibrium. All three carrageenans show qualitatively similar spectra, with *G′* one order of magnitude larger than the shear loss modulus *G*″. These spectra are comparable to those reported in some references listed in Table 1 and Table 2 for both Iota and Kappa in various salt conditions [29,30,32,35,38]. 

The similarity between the mechanical spectra of Iota and Kappa2 is reflected in the EM pictures of the corresponding gels. The latter were collected from the rheometer, frozen and fractured with an Alto 2500 (Gatan Inc., Pleasanton, CA, USA) cryo preparation chamber and imaged with a JEOL JSM 6301F scanning electron microscope (Tokyo, Japan). However, qualitative similarities in both structure and mechanical spectra cannot explain the significant larger elasticity of Kappa2 (see the values of *G′* shown in the insets to Figure 3b,c, which are of the order of 4.4 kPa for Kappa2 against 0.3 kPa for Iota). In contrast to this, one might assign the larger elasticity exhibited by the Kappa gel (*G*_0_ = 24 kPa in Figure 3a) to the denser network with smaller pores in the corresponding EM picture. Thus, network connectivity seems to be related to the larger elasticity of Kappa gels, whereas differences in the elasticities of Kappa2 and Iota gels can only be rationalized so far by differences in the *E_bend_* of filaments (see Equations (4b) and (6b) in Table 3) or by the enthalpic or entropic natures of the corresponding networks (see Equations (2c) and (2d) in Table 3).

**Figure 4:**
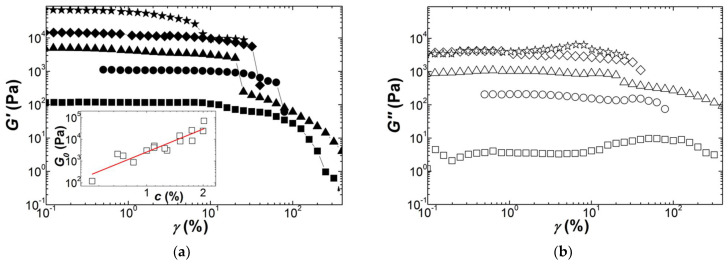
Response of Kappa gels (squares: 0.55 wt.%; circles: 0.75 wt.%; triangles 1.1 wt.%; diamonds: 1.5 wt.%; stars: 2 wt.%) to a sweep in the dynamic shear strain *γ* at 1 Hz and 20 °C: (**a**) storage modulus *G′*; (**b**) loss modulus *G*″. Inset: concentration dependence of the storage modulus *G*_0_ measured in the linear regime; the line is a power law fit to the data, returning an exponent of *n* = 3.5 ± 0.4.

Following the record of the mechanical spectra, an oscillatory strain sweep was performed at a frequency of 1 Hz. Thirty cycles were applied for each stepped strain amplitude, which is enough to perform a full Fourier transform analysis of the nonlinear oscillatory stress response [62]. However, the nonlinear rheological response of gels is here analyzed with only the strain dependence of the storage modulus *G′*(*γ*) and the loss modulus *G*″(*γ*). This is because the nonlinear index measured by the rheometer showed that more than 70% of the nonlinearity was contained in the fundamental component of the non-sinusoidal stress response. In addition, a preliminary analysis of *G′*(*γ*) with the equations *G*(*γ*) listed in Table 3 is presented here, whereas further *G*(*γ*) computation in the Fourier space will be detailed elsewhere to give a full report on the Fourier transform rheology of Kappa, Iota and Kappa2 gels. Figure 4 presents the strain dependence of the shear moduli measured during the dynamic strain sweeps performed on Kappa gels prepared at different carrageenan concentrations. The strain for onset of nonlinear behavior *γ_NL_* is shifted to smaller strains as the concentration in Kappa is increased, spanning a range between 10% and 1% for the range of concentrations tested. This is in harmony with the values for *γ_NL_* reported in a few studies [30,32,63], which also document the abrupt decay seen in Figure 4 for both G′ and G″ beyond *γ_NL_*. A strain softening, characterized by a smoother drop in *G′* coinciding with a local maximum in G″, is also found at the smallest and largest concentrations tested before a more acute drop in the shear moduli at greater strains. The inset to Figure 4a reports the concentration dependence of *G*_0_ for all gels tested and extracted from the plateau in *G′*(*γ*) at small strains. The fit of the *G*_0_ data to a power law returns an exponent of *n* = 3.5 ± 0.4. The latter is in fair agreement with those figures listed in Table 4. 

The scatter in the *G*_0_ data is indicative of the difficulty of achieving reproducible experiments, which calls for additional nonlinear testing with different protocols and shearing geometries. 

**Figure 5:**
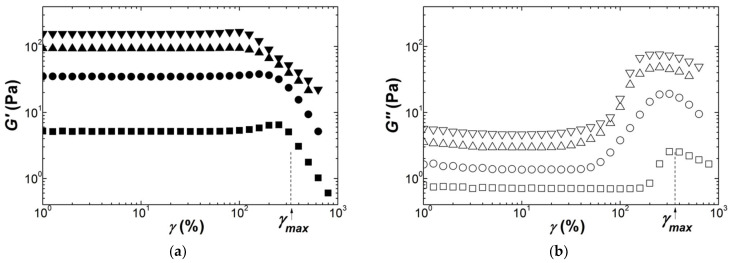
Response of Iota gels (squares: 0.3 wt.%; circles: 0.7 wt.%; up triangles 1.2 wt.%; down triangles: 1.7 wt.%) to a sweep in the dynamic shear strain *γ* at 1 Hz and 20 °C: (**a**) storage modulus *G′*; (**b**) loss modulus *G*″. *γ_max_* signals the strain where *G*″ passes through a local maximum for the gel with 0.3 wt.% Iota.

**Figure 6:**
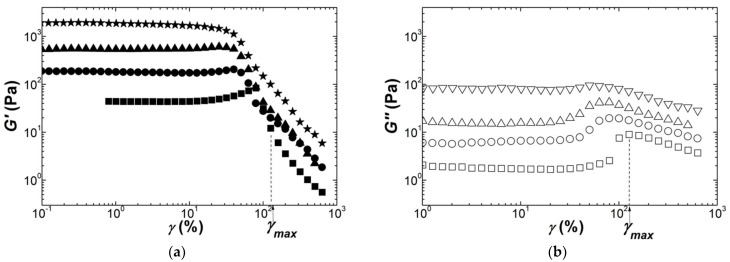
Response of Kappa2 gels (squares: 0.5 wt.%; circles: 0.75 wt.%; up triangles 1.1 wt.%; stars: 1.5 wt.%) to a sweep in the dynamic shear strain *γ* at 1 Hz and 20 °C: (**a**) storage modulus *G′*; (**b**) loss modulus *G*″. *γ_max_* signals the strain where *G*″ passes through a local maximum for the gel with 0.5 wt.% Kappa2.

Figure 5 and Figure 6 show the nonlinear rheological behaviors of Iota and Kappa2 gels. 

For both carrageenans, the storage modulus *G′* exhibits strain hardening at lower strains before a decrease starting at *γ_max_*, where a maximum in the loss modulus G″ is located. A qualitatively similar strain hardening has recently been reported for an Iota gel (2 wt.% with no salt) [38], whereas strain softening has been documented when Iota is gelled in the presence of salts [32,63]. Indeed, the strain hardening is less evident for the gels formed at higher carrageenan concentrations for both Iota and Kappa2. 

**Figure 7:**
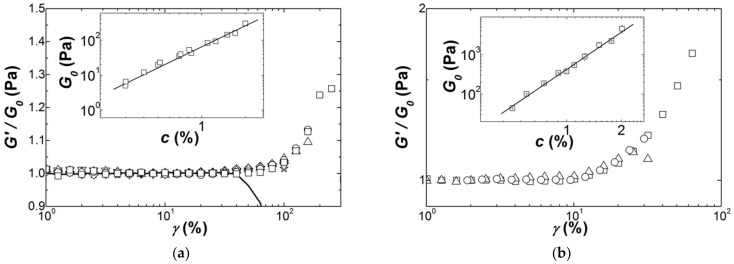
Strain dependence of the shear storage modulus *G′* scaled by the linear shear modulus *G*_0_ of the corresponding gels: (**a**) Iota gels with 0.3 wt.% (squares), 0.5 wt.% (circles), 0.7 wt.% (up triangles), 0.8 wt.% (down triangles), 1.1 wt.% (diamonds), 1.25 wt.% (stars) and 2 wt.% (line); (**b**) Kappa2 gels with 0.5 wt.% (squares), 0.75 wt.% (circles) and 1.1 wt.% (triangles). Insets present the concentration dependence of *G*_0_ and lines are power law fits to the data.

More interesting is the fact that the strain hardening exhibits a concentration scaling. This is shown in Figure 7, where *G′*(*γ*) curves are scaled by the linear shear modulus *G*_0_. The scaled curves highlight the power law dependence *G*_0_ = *Ac^n^* which is displayed in the insets to Figure 7, with *n* = 2.01 ± 0.08 for Iota and *n* = 3.1 ± 0.1 for Kappa2. This concentration scaling is in harmony with the theoretical treatments reviewed in Table 3: the structural and elastic features of worm-like filaments remain the same for all concentrations (see Equations (2c), (2d) and (4b)). 

Therefore, the comparison of the theories designed for networks of filaments with the elastic data presented in Figure 7 can now be performed.

## 5. Rationalizing Structural and Elastic Results by Mechanical Models

**Figure 8:**
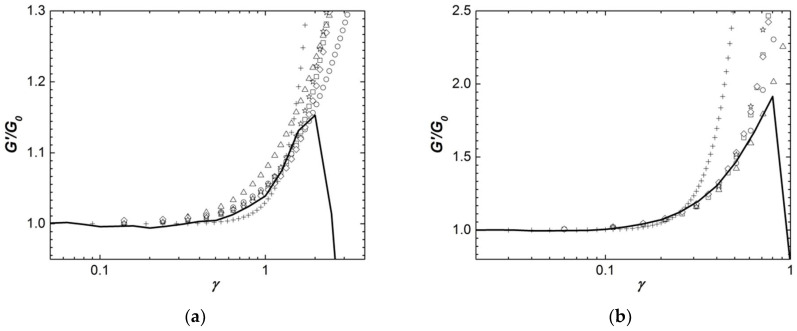
Strain dependence of the shear storage modulus *G′* scaled by the linear shear modulus *G*_0_, plotted as lines and measured with: (**a**) Iota gel with 0.3 wt.%; (**b**) Kappa2 gel with 0.5 wt.%. The symbols represent the following equations computed with the parameters listed in Table 5: Equation (1a), squares; Equation (2a), circles; Equation (3a), triangles; Equation (4a), diamonds; Equation (5), stars; Equation (6a), crosses.

Figure 8 compares the strain dependence of the shear storage modulus *G′*(*γ*) of two representative Iota and Kappa2 gels with the equations listed in Table 3 and computed using the parameters displayed in Table 5. 

Gels formed with 0.3 wt.% Iota and 0.5 wt.% Kappa2 were selected, as they present the widest experimental window for capturing the strain hardening, and thus offer a better comparison with the theoretical predictions. 

Figure 8 suggests overall that the experimental data cannot be reproduced by a network of rod-like filaments without crosslinks, in spite of the introduction of an additional term to Equation (6a). It has been shown elsewhere that a Gaussian network with linear shear elastic modulus *G_e_* is needed to delay the strain hardening towards larger strain amplitudes [64]. Equation (6a) gives a too steep hardening which cannot accommodate the nonlinear elastic properties of the carrageenan gels tested here. The fact that this theory cannot describe the networks built by Iota and Kappa2 was expected since the power law exponents *n* computed from the concentration dependence of *G*_0_ largely differ from 5 and also because structural analysis has long established the existence of crosslinks in Iota gels and pre-gels [19]. Similarly, Figure 8a indicates that Equation (3a) cannot reproduce the experiments with Iota gels. In contrast to this, Figure 8b suggests that the Kappa2 gel is made of rod domains which are 100 times larger than the coils connecting them (see parameter *L*/*R*_0_ in Table 5). 

The remaining equations give a fair account of the nonlinear elastic behavior of Iota and Kappa2 gels. They all suggest that the wormlike strands in Kappa2 gels are nearly six times more extended than those in Iota gels (compare, e.g., parameters *l_max_*/*l_o_*, *β* or *x* in Table 5), and this inherently explains why nonlinearity is reached at smaller strains for Kappa2 when compared to Iota. Based on the established differences in the persistence lengths of Kappa and Iota [12], one can conjecture that the copolymeric nature of Kappa2 entails a persistence length between those of Kappa and Iota, giving rise to self-assembly into straighter filaments than in Iota. The structural information conveyed by the pictures displayed in Figure 3 is that both Kappa2 and Iota gels exhibit similar network density, i.e., they show similar *ε* values. Taking on board the theoretical meaning of parameter *x* in Equation (5), this suggests that *L_c_* is larger for Iota, as expected from the above arguments about persistence length. Interestingly, parameter *e* in Equation (5) is found to be 0.55 for both types of carrageenans, thus suggesting that self-assembly occurs with filaments of similar elasticity (see Table 3), resulting from a balance between their bending rigidity and their conformation [51].

Two issues need to be noted here in the rationalization of the nonlinear rheological data by some strain hardening equations. The fractal dimension *d_f_* computed from the nonlinear elastic behavior of Kappa2 gels (Equation (2a)) does not comply with the power law exponent *n* = 3.1 ± 0.1 inferred from their linear elastic behavior. *n* values computed from *d_f_* using either the enthalpic or entropic hypothesis are significantly smaller (2.3 and 1.65, respectively; see Table 5). The same issue arises for Iota gels but is less dramatic since the entropic *n* estimated from *d_f_* is 2.4, which is closer to the value (*n* = 2.01 ± 0.08) estimated from the concentration scaling of *G*_0_. Note here that the more entropic nature of the Iota gels complies with the looser nature of the network inferred from the data analysis with Equations (1a), (4a) and (5), and the fact that a Gaussian elasticity *G_e_* needs to be added to Equation (4a) to reproduce its elastic behavior [50]. However, Kappa2 gels require larger contributions from Gaussian elasticity to reproduce the data with Equation (4a). This is at odds with the strain hardening attributed to the presence of more stretched filaments between crosslinks. In addition, the scaling of *G*_0_ with the concentration in Kappa2 is significantly larger than the quadratic prediction of Equation (4b). Thus, Gaussian chains are needed to describe the larger linear elasticity of Kappa2 gels, whereas straighter filaments (see parameter *β*) are required to impart strain hardening. This is, indeed, the second issue of the data analysis presented here. Additional structural parameters in Equation (4a) are thus needed to better describe the elastic behavior of networks whose elastic nature is between entropic and enthalpic [24,50]. Note here that Equation (5) partially helps in settling this issue to some extent. It incorporates the filament flexibility (parameter *e*) to give a more consistent description of the nonlinear elasticity of both Kappa2 and Iota, which only differ by their structural parameter *x*. 

## 6. Conclusions and Perspectives

The literature on the structure–gel elastic properties relationships reviewed here gives ample evidence for the filamentous nature of the networks responsible for the elasticity of carrageenan gels. This structure explains the power law dependence of the gel linear elastic modulus *G*_0_ on the carrageenan concentration and the strain hardening behavior of Iota gels. Although not sufficiently documented in the literature, the strain hardening is established in the present study and rationalized by theoretical models. The latter explain the quadratic concentration scaling of the strain hardening which stems from the rod-like shape of the filaments (with fractal dimensions of the order of 1.7) imparting more enthalpic elasticity than entropic elasticity to the network.

The picture for Kappa gels is much less clear, as difficulties in the rheological testing of these material may explain the scatter in the power law exponents reported in the reviewed literature to describe the concentration dependence of *G*_0_. Due to its larger persistence length, Kappa self-assemble in straighter and more connected strands when compared with Iota. However, a review of the literature indicates that both systems show nearly identical strand lengths (of the order of 100 nm) and radii (of the order of 1 nm). Mesh sizes in the network are also reported to be nearly identical. Therefore, the superior elasticity of Kappa gels has been assigned to the greater stiffness of Kappa strands (or filaments), as was suggested by authors who established that Kappa forms straighter strands [26,32]. Several recent theoretical treatments of filamentous networks’ elasticities, briefly reviewed here, suggest that in this case Kappa gels should be strain hardening. However, strain thinning followed by an abrupt gel yielding is consistently reported in the limited sample of the literature reviewed here. The present study confirms this high strain behavior.

A way to bypass the rheometrical issues associated with the water syneresis and high elasticity of Kappa gels could be to study less rigid Kappa2 gels with varied compositions in Kappa and Iota blocks in the copolymer. A preliminary experimental study of the nonlinear elastic properties of a specific Kappa2 has been presented here. The results show that this Kappa2 (i) forms stronger gels than Iota, (ii) shows a stronger concentration scaling of its strain hardening behavior and (iii) hardens at lower shear strains. The analysis of the results by the predictions of a series of strain hardening theories suggests that models built from the elasticity of wormlike chains crosslinked in a network reproduce more closely the set of rheological data. This theoretical treatment suggests that Kappa2 gels are made of straighter strands arranged in a denser network than in Iota gels but with similar strand stiffness. 

An additional nonlinear elastic investigation is still needed to relate the elastically relevant structural features with the nanoscale structural characteristics of Kappa2 gels. In this sense, the calls made over a decade ago in the “carrageenan community” for further investigation of the high strain behavior of carrageenan gels [19,25] are still appropriate, though for a different reason: recent progress in the theoretical understanding of the strain hardening of filamentous networks has been made. Future studies, with Kappa2 containing varying amounts of Kappa blocks up to a full Kappa, should take into account the following issues:

- Effects of stress build-up in the network and the systematic study of normal stresses under large strains. A volume change often accompanies the sol–gel transition during cooling. The latter triggers the build-up of stresses within the gel (pre-stress), which can be monitored or even removed by controlling normal stresses [65]. In addition, such pre-stress influences the phase diagram of filamentous networks [51] and thus their nonlinear elastic response, which can show negative or positive Poynting effects. Thus, measuring normal stresses generated by gel-setting and large strains is important to help distinguish between entropic or enthalpic elasticity in filamentous networks [51] and to clarify the absence of strain hardening in Kappa gels.
- In rheometer structural characterization during carrageenan gel build-up, e.g., with in situ birefringence measurements [66], should be re-visited to avoid differences in the thermal history of gels prepared for structural characterization and rheology. Further, such rheo-optical measurements will help to assign structural changes to large strain behavior.
- Nonlinear elasticity should be studied with additional methods which incorporate the time of deformation [67], not restricting studies to the dynamic oscillatory testing reviewed here. In particular, such time needs to be taken into account during the thixotropic study of carrageenan gels, a topic of industrial interest not touched on here.
- Most importantly of all, and as pointed out by Picullel [19], efforts should be made for studying model carrageenans, i.e., with established disaccharide composition and a single type of counter-ion. Thus, the tailored extraction of carrageenans from selected seaweeds seems preferable to converting commercial samples into a single cation form, since such processes are known to degrade the polysaccharide [68].

## Figures and Tables

**Table 4:** Exponents *n* of the power law equation *G*_0_ = *Ac^n^* describing the dependence of the linear shear elastic modulus *G*_0_ on the carrageenan concentration *c* in gels formed under the conditions detailed in the column “Sample”. The values of constant *A* used in the fitting of the power equation to the data are not listed.

Sample				*n*	Ref.
Carrageenan Form ^1^	Salt	Temperature (°C)	Concentration Range (wt.%)		
Kappa					
Com.	No salt	10	0.8–3.5	2.7	[29]
Com.	No salt	40	0.8–2.5	2.7	[29]
Na	KCl + NaCl	15	0.03–0.2	2.1	[52]
K	No salt	20	0.7–1.4	7.7 ± 0.2 ^2^	[53]
Na	KCl	5	0.25–3	3.2 ± 0.3 ^2^	[54]
Com.	KCl	25	1.1–2	2.4 ± 0.1 ^2^	[55]
Com.	NaCl	20	0.5–2	3.5 ± 0.4	t.w. ^4^
Iota					
Na	KCl	5	0.5–3	3.4 ± 0.3 ^2^	[54]
Com.	No salt	25	7–7	3.2	[56]
Com.	KCl	25	1–2.5	2.1	[56]
Na	NaCl	n.a. ^3^	2–3	10 ± 1 ^2^	[57]
K	KCl	n.a. ^3^	1–2	3.7 ± 0.4 ^2^	[57]
Ca	CaCl_2_	n.a. ^3^	0.25–1.5	1.6 ± 0.3 ^2^	[57]
Com.	NaCl	20	0.3–2	2.01 ± 0.08	t.w. ^4^

^1^ Com.: commercial carrageenan used as received; Na: sodium form; K: potassium form; Ca: calcium form. ^2^ Computed after re-plotting original data and fitting to a power law. ^3^ n.a.: not available. ^4^ t.w.: this work (see Section 4).

**Table 5:** Numerical values of the parameters used in the equations presented in Section 3 to compute *G*(*γ*)/*G*_0_ data presented in Figure 8.

Iota	Kappa2	Structural Parameters	Equations
4.7	0.8	*l_max_*/*l*_0_	(1a)
1.75	1.18	*d_f_*	(2a)
3.8	2.3	$n=\frac{3+d_{f}}{3-d_{f}}$	(2c)
2.4	1.65	$n=\frac{3}{3-d_{f}}$	(2d)
0.01	100	*L*/*R*_0_	(3)
0.07	0.5	*β*	(4a)
0.1	2	*G_e_*/*G*_0_	(4a) ^1^
0.15	0.15	*e*	(5)
0.09	0.55	x	(5)
2.5	0.01	*G_e_*/*G*_0_	(6a) ^1^

^1^*G_e_* is the linear elastic modulus of the Gaussian network, added to the original equation to describe the range of smaller strains [50,64].
